# Supplementary material for: A Likelihood Approach for Real-Time Calibration of Stochastic Compartmental Epidemic Models
Source: PLoS Comput Biol. 2017 Jan 17;13(1):e1005257. doi: 10.1371/journal.pcbi.1005257 (PMC5240920; doi:10.1371/journal.pcbi.1005257)
Supplement: S1 File — (TAR.GZ) [file pcbi.1005257.s014.tar.gz › HSPH_Online-SI-Revision/MSS10/n10-severe/n10-severe_NEW_table8.pdf]

1	R0	{1.68952, 11.8402, 1.}	{1.69066, 2.19961, 0.}	{{1.67287, 2.12856, 0.}, {1.70301, 2.24898, 0.}}	{0.87523, 81.4226, 100.}	inf	198.	26.3829	{8.4262, 41.8523}	86.6753	0.958367	newly	99.	46.61	{9., 79.}	53.2828	0.432426	Reff	0.571396	0.600795	{0.593084, 0.607378}	5.14517	ar	6872.	6484.35	{6434., 6529.}	5.64101
2	R0	{1.71739, 9.62661, 0.1}	{1.81391, 11.3493, 1.}	{{1.81391, 11.3493, 1.}, {1.81391, 11.3493, 1.}}	{5.62029, 17.8946, 900.}	inf	184.	154.46	{141.009, 166.925}	16.0543	0.076789	newly	116.	84.48	{73., 96.}	27.1724	0.139949	Reff	0.569658	0.607033	{0.60474, 0.609455}	6.5609	ar	6874.	6875.71	{6820., 6947.}	0.613471
3	R0	{1.48936, 6.23975, 0.1}	{1.47651, 5.4396, 3.}	{{1.47651, 5.4396, 3.}, {1.47651, 5.4396, 3.}}	{0.86282, 12.8234, 2900.}	inf	38.	25.2948	{15.9741, 33.0048}	34.1804	0.198258	newly	40.	26.3	{15., 36.}	35.4	0.211412	Reff	0.649957	0.646225	{0.644937, 0.647533}	0.574196	ar	5666.	5666.58	{5634., 5703.}	0.38581
4	R0	{1.62394, 7.51135, 1.}	{1.58886, 7.29751, 1.}	{{1.58886, 7.29751, 1.}, {1.58886, 7.29751, 1.}}	{2.15991, 2.84689, 0.}	inf	86.	72.8522	{61.1837, 84.9575}	15.9483	0.0779504	newly	66.	57.43	{47., 67.}	15.6818	0.0762675	Reff	0.601507	0.590604	{0.588605, 0.592409}	1.81261	ar	6417.	6391.1	{6343., 6448.}	0.596229
5	R0	{1.53632, 8.57652, 1.}	{1.72632, 9.16352, 3.08}	{{1.58672, 2.78994, 0.}, {1.85253, 15.8132, 4.}}	{12.3675, 74.1584, 230.}	inf	148.	276.935	{31.97, 541.904}	160.497	0.55095	newly	112.	139.92	{52., 226.}	64.9643	0.258073	Reff	0.610687	0.661009	{0.646702, 0.671473}	8.24024	ar	6260.	6565.33	{5959., 7221.}	9.16885
6	R0	{1.47166, 5.7434, 0.1}	{1.47686, 4.99072, 2.13}	{{1.47488, 4.06661, 0.}, {1.47651, 5.4396, 3.}}	{0.353155, 13.1051, 2088.}	inf	52.	34.5391	{18.6955, 50.5934}	35.866	0.22249	newly	51.	38.05	{22., 55.}	29.9804	0.173268	Reff	0.657979	0.662882	{0.660127, 0.664868}	0.746706	ar	5650.	5575.51	{5534., 5629.}	1.3492
7	R0	{1.48179, 8.87369, 0.1}	{1.41695, 7.40677, 3.}	{{1.41695, 7.40677, 3.}, {1.41695, 7.40677, 3.}}	{4.37566, 16.5311, 2900.}	inf	96.	91.4497	{78.7109, 106.185}	10.404	0.0472953	newly	76.	74.92	{60., 88.}	12.5263	0.0563441	Reff	0.721187	0.690275	{0.68816, 0.692039}	4.28625	ar	5233.	5316.29	{5240., 5403.}	1.64361
8	R0	{1.53669, 6.47807, 0.1}	{1.54465, 5.30296, 4.}	{{1.54465, 5.30296, 4.}, {1.54465, 5.30296, 4.}}	{0.518036, 18.1399, 3900.}	inf	51.	24.3061	{13.4546, 34.3037}	52.3409	0.359	newly	34.	25.57	{13., 37.}	30.4412	0.187316	Reff	0.629736	0.637121	{0.635519, 0.638748}	1.1728	ar	5934.	5915.31	{5880., 5951.}	0.469329
9	R0	{1.57227, 6.20989, 0.1}	{1.54511, 5.30092, 4.}	{{1.54465, 5.30296, 4.}, {1.54465, 5.30296, 4.}}	{1.75059, 14.6375, 3900.}	inf	66.	37.0182	{21.7772, 48.3226}	43.9118	0.266964	newly	57.	38.74	{23., 53.}	32.6316	0.188435	Reff	0.625135	0.618812	{0.616884, 0.620984}	1.04141	ar	6136.	6052.83	{6009., 6102.}	1.36783
10	R0	{1.54936, 6.50938, 0.1}	{1.59093, 5.09139, 3.98}	{{1.59034, 5.09935, 4.}, {1.59034, 5.09935, 4.}}	{2.68334, 21.7837, 3880.}	inf	32.	16.0617	{6.59724, 27.1399}	49.807	0.353242	newly	31.	17.35	{8., 31.}	45.5806	0.319266	Reff	0.582559	0.600727	{0.598726, 0.602009}	3.11862	ar	6275.	6247.21	{6224., 6269.}	0.48749
11	R0	{1.42179, 3.18601, 0.1}	{1.44006, 2.61125, 1.01}	{{1.43965, 2.60601, 1.}, {1.43965, 2.60601, 1.}}	{1.28509, 18.0403, 910.}	inf	5.	5.41117	{1.50751, 9.55733}	53.8473	0.249319	newly	7.	9.85	{3., 18.}	75.8571	0.27899	Reff	0.665398	0.67389	{0.673041, 0.674257}	1.27622	ar	5322.	5330.84	{5318., 5349.}	0.202931
12	R0	{1.48035, 6.46183, 0.1}	{1.47684, 5.43316, 3.}	{{1.47651, 5.4396, 3.}, {1.47651, 5.4396, 3.}}	{0.276745, 15.9192, 2900.}	inf	58.	40.9501	{30.2463, 52.0692}	29.4344	0.164388	newly	54.	42.27	{29., 56.}	23.9074	0.130098	Reff	0.636995	0.638004	{0.636164, 0.63944}	0.212363	ar	5793.	5746.49	{5701., 5798.}	0.889177
13	R0	{1.75755, 8.53854, 1.}	{1.67405, 7.28757, 1.}	{{1.67405, 7.28757, 1.}, {1.67405, 7.28757, 1.}}	{4.75096, 14.6508, 0.}	inf	105.	94.9853	{80.9571, 108.024}	10.9014	0.0517056	newly	83.	73.88	{61., 87.}	13.253	0.0641451	Reff	0.574719	0.549091	{0.546891, 0.551332}	4.45925	ar	6832.	6834.91	{6794., 6881.}	0.384514
14	R0	{1.47355, 4.89451, 0.1}	{1.4965, 5.05311, 3.}	{{1.47651, 4.79544, 3.}, {1.50982, 5.4396, 3.}}	{1.55723, 5.66913, 2900.}	inf	18.	21.2331	{10.1712, 32.6878}	41.2476	0.17617	newly	20.	22.77	{11., 36.}	40.65	0.185909	Reff	0.635542	0.644962	{0.634812, 0.652289}	1.53609	ar	5710.	5726.03	{5690., 5761.}	0.453765
15	R0	{1.62071, 5.98665, 0.1}	{1.65865, 5.3704, 5.}	{{1.65865, 5.3704, 5.}, {1.65865, 5.3704, 5.}}	{2.34101, 10.2938, 4900.}	inf	12.	20.6671	{12.1822, 28.7802}	77.5933	0.245151	newly	11.	20.99	{12., 30.}	96.2727	0.286075	Reff	0.547962	0.559352	{0.558007, 0.560739}	2.07866	ar	6635.	6653.46	{6631., 6674.}	0.319819
16	R0	{1.67268, 7.7256, 1.}	{1.77826, 9.06807, 1.}	{{1.77826, 9.06807, 1.}, {1.77826, 9.06807, 1.}}	{6.31183, 17.3769, 0.}	inf	38.	7.10532	{2.36648, 11.5521}	81.3018	0.800403	newly	29.	4.56	{1., 8.}	84.2759	0.906199	Reff	0.519367	0.557643	{0.556783, 0.55848}	7.36963	ar	6947.	6873.77	{6859., 6890.}	1.05412
17	R0	{1.67805, 11.1417, 1.}	{1.6082, 8.77064, 4.}	{{1.6082, 8.77064, 4.}, {1.6082, 8.77064, 4.}}	{4.16274, 21.2809, 300.}	inf	310.	287.809	{266.472, 309.187}	7.46295	0.0342342	newly	183.	196.66	{177., 218.}	9.08197	0.0371511	Reff	0.643364	0.620152	{0.616541, 0.62348}	3.60803	ar	6625.	6546.59	{6451., 6637.}	1.35804
18	R0	{1.66743, 9.99046, 0.1}	{1.61188, 8.7843, 3.}	{{1.61188, 8.7843, 3.}, {1.61188, 8.7843, 3.}}	{3.33157, 12.0732, 2900.}	inf	216.	114.792	{104.628, 125.503}	46.8557	0.275907	newly	133.	77.12	{66., 87.}	42.015	0.23939	Reff	0.574763	0.571928	{0.570192, 0.573549}	0.499538	ar	6899.	6597.79	{6541., 6642.}	4.366
19	R0	{1.70787, 9.85478, 0.1}	{1.84021, 13.0803, 2.}	{{1.84021, 13.0803, 2.}, {1.84021, 13.0803, 2.}}	{7.7489, 32.7309, 1900.}	inf	121.	382.188	{365.741, 396.157}	215.858	0.499312	newly	78.	181.67	{166., 199.}	132.91	0.366246	Reff	0.56428	0.559942	{0.557346, 0.562553}	0.770272	ar	6867.	7369.85	{7309., 7429.}	7.3227
20	R0	{1.53809, 10.5848, 1.}	{1.47923, 2.40449, 2.6}	{{1.43965, 2.26472, 1.}, {1.48799, 2.60601, 3.}}	{3.8267, 77.2836, 160.}	inf	283.	65.8127	{36.7295, 95.3187}	76.7446	0.664123	newly	160.	121.64	{68., 175.}	28.3625	0.168475	Reff	0.669684	0.676179	{0.660008, 0.685087}	1.53599	ar	6108.	5557.27	{5466., 5651.}	9.01654
21	R0	{1.59925, 6.74479, 0.1}	{1.65865, 5.3704, 5.}	{{1.65865, 5.3704, 5.}, {1.65865, 5.3704, 5.}}	{3.7143, 20.3771, 4900.}	inf	59.	34.2424	{24.2723, 45.2614}	41.962	0.248891	newly	39.	35.	{23., 46.}	19.8974	0.0972255	Reff	0.593482	0.619632	{0.617776, 0.621204}	4.40622	ar	6411.	6320.78	{6282., 6366.}	1.41101
22	R0	{1.38779, 4.40977, 0.1}	{1.416, 4.94736, 2.}	{{1.41571, 4.94504, 2.}, {1.41571, 4.94504, 2.}}	{2.033, 12.3742, 1902.}	inf	49.	44.0884	{27.4906, 58.4463}	21.327	0.106028	newly	54.	50.21	{29., 71.}	24.0185	0.117421	Reff	0.682793	0.69737	{0.694701, 0.699664}	2.13489	ar	5154.	5171.94	{5112., 5241.}	0.849437
23	R0	{1.53657, 7.50352, 1.}	{1.48067, 3.13663, 1.94}	{{1.4804, 3.12918, 2.}, {1.4804, 3.12918, 2.}}	{3.63766, 58.1978, 98.}	inf	141.	40.926	{21.1769, 59.09}	70.9745	0.574277	newly	110.	62.39	{28., 92.}	43.3909	0.283336	Reff	0.661647	0.652397	{0.649032, 0.655198}	1.49836	ar	5928.	5663.96	{5600., 5723.}	4.45412
24	R0	{1.71131, 6.24526, 0.1}	{1.69756, 6.15907, 2.}	{{1.69756, 6.15907, 2.}, {1.69756, 6.15907, 2.}}	{0.803188, 1.3801, 1900.}	inf	47.	29.474	{18.8424, 37.7537}	37.376	0.218287	newly	36.	26.63	{16., 38.}	29.0278	0.164796	Reff	0.538036	0.53669	{0.535041, 0.538258}	0.28777	ar	6924.	6871.7	{6842., 6900.}	0.772097
25	R0	{1.4412, 3.07729, 0.1}	{1.4804, 3.12918, 2.}	{{1.4804, 3.12918, 2.}, {1.4804, 3.12918, 2.}}	{2.71978, 1.6862, 1900.}	inf	2.	6.50843	{2.33033, 11.6823}	230.001	0.461314	newly	2.	9.88	{3., 18.}	398.	0.621327	Reff	0.603863	0.619619	{0.618801, 0.620201}	2.60925	ar	5810.	5826.62	{5811., 5848.}	0.287435
26	R0	{1.4159, 4.93858, 0.1}	{1.41603, 4.93564, 1.98}	{{1.41571, 4.94504, 2.}, {1.41571, 4.94504, 2.}}	{0.0354089, 0.318308, 1882.}	inf	48.	34.7668	{24.1687, 45.4452}	29.2197	0.161924	newly	55.	39.76	{28., 54.}	29.0182	0.162996	Reff	0.683172	0.68511	{0.68329, 0.68645}	0.302263	ar	5255.	5228.79	{5182., 5271.}	0.686775
27	R0	{1.57465, 7.05414, 0.1}	{1.58886, 7.29751, 1.}	{{1.58886, 7.29751, 1.}, {1.58886, 7.29751, 1.}}	{0.902704, 3.45003, 900.}	inf	116.	113.525	{98.4653, 126.607}	8.19066	0.0365812	newly	88.	91.43	{75., 106.}	10.7841	0.0451722	Reff	0.599784	0.605592	{0.603312, 0.607936}	0.968265	ar	6359.	6347.54	{6281., 6414.}	0.643183
28	R0	{1.41422, 4.62402, 0.1}	{1.45826, 4.02718, 0.}	{{1.44762, 4.00571, 0.}, {1.47488, 4.06661, 0.}}	{3.11437, 12.9074, 100.}	inf	11.	15.0148	{3.83987, 24.1509}	64.5614	0.248283	newly	15.	19.83	{5., 35.}	60.7333	0.240944	Reff	0.643046	0.662487	{0.656434, 0.670965}	3.02334	ar	5485.	5488.	{5451., 5524.}	0.423701
29	R0	{1.41901, 3.63017, 0.1}	{1.53501, 4.02511, 0.}	{{1.53501, 4.02511, 0.}, {1.53501, 4.02511, 0.}}	{8.17462, 10.8793, 100.}	inf	5.	8.76335	{3.18838, 14.479}	91.2008	0.271436	newly	11.	11.02	{4., 18.}	44.7273	0.229758	Reff	0.571577	0.617724	{0.616828, 0.618567}	8.07355	ar	5979.	5990.65	{5972., 6012.}	0.243686
30	R0	{1.60719, 4.72984, 0.1}	{1.62768, 4.37796, 3.34}	{{1.61691, 4.30394, 2.}, {1.64953, 4.41442, 4.}}	{1.27459, 7.43955, 3240.}	inf	1.	10.8432	{3.79656, 17.8977}	984.319	0.973149	newly	3.	12.27	{4., 19.}	312.333	0.566879	Reff	0.566374	0.57199	{0.567429, 0.579982}	0.991579	ar	6479.	6500.85	{6481., 6521.}	0.339096
31	R0	{1.38175, 6.26468, 0.1}	{1.45757, 4.34113, 0.22}	{{1.43965, 2.60601, 0.}, {1.47608, 6.00596, 1.}}	{5.48698, 30.7048, 278.}	inf	50.	36.181	{15.6049, 51.9416}	32.4333	0.216687	newly	41.	45.11	{22., 63.}	32.6585	0.155538	Reff	0.648593	0.686199	{0.677082, 0.696521}	5.79802	ar	5397.	5366.07	{5298., 5433.}	0.96776
32	R0	{1.4947, 8.8847, 1.}	{1.46362, 3.47829, 0.35}	{{1.43965, 2.60601, 0.}, {1.47608, 3.83841, 1.}}	{2.07943, 60.8508, 71.}	inf	106.	38.4706	{18.9059, 57.0469}	63.707	0.476793	newly	69.	59.21	{30., 84.}	28.2464	0.153639	Reff	0.721193	0.716073	{0.704332, 0.72424}	0.914247	ar	5407.	5197.72	{5125., 5286.}	3.87054
33	R0	{1.55891, 8.28978, 1.}	{1.59539, 3.01252, 3.8}	{{1.58672, 2.78994, 4.}, {1.61068, 3.05492, 4.}}	{2.33979, 63.6598, 290.}	inf	118.	44.3418	{27.2021, 61.0103}	62.4222	0.44703	newly	80.	71.98	{39., 100.}	26.775	0.13571	Reff	0.628553	0.655015	{0.648852, 0.662871}	4.21005	ar	6239.	5979.6	{5911., 6048.}	4.15772
34	R0	{1.60764, 10.4729, 0.1}	{1.67287, 2.12856, 0.}	{{1.67287, 2.12856, 0.}, {1.67287, 2.12856, 0.}}	{4.05763, 79.6756, 100.}	inf	168.	28.1573	{6.74132, 49.8754}	83.2397	0.8732	newly	102.	53.63	{14., 94.}	49.0098	0.384521	Reff	0.600936	0.648713	{0.645073, 0.652222}	7.95054	ar	6495.	6172.93	{6108., 6245.}	4.95874
35	R0	{1.48527, 5.74485, 1.}	{1.47651, 5.4396, 3.}	{{1.47651, 5.4396, 3.}, {1.47651, 5.4396, 3.}}	{0.589825, 5.31342, 200.}	inf	59.	34.1161	{23.2234, 44.8218}	42.1761	0.258445	newly	58.	35.41	{21., 49.}	39.1552	0.241978	Reff	0.640597	0.640493	{0.638875, 0.642075}	0.163161	ar	5811.	5722.24	{5685., 5764.}	1.5333
36	R0	{1.61457, 9.93364, 1.}	{1.56097, 8.77207, 2.}	{{1.56097, 8.77207, 2.}, {1.56097, 8.77207, 2.}}	{3.31968, 11.6933, 100.}	inf	204.	142.132	{126.318, 155.802}	30.3277	0.158241	newly	131.	98.32	{84., 113.}	24.9466	0.127437	Reff	0.638401	0.626866	{0.624727, 0.629278}	1.80692	ar	6338.	6203.31	{6147., 6269.}	2.12543
37	R0	{1.47831, 7.08139, 0.1}	{1.44485, 6.00596, 0.}	{{1.44485, 6.00596, 0.}, {1.44485, 6.00596, 0.}}	{2.26358, 15.1867, 100.}	inf	93.	49.9219	{33.1283, 62.9955}	46.3205	0.282533	newly	79.	48.65	{30., 63.}	38.4177	0.225843	Reff	0.683866	0.67461	{0.672717, 0.677013}	1.35344	ar	5530.	5430.2	{5371., 5494.}	1.8311
38	R0	{1.73445, 9.8858, 0.1}	{1.69397, 2.21285, 0.}	{{1.67287, 2.12856, 0.}, {1.70301, 2.24898, 0.}}	{2.33379, 77.6158, 100.}	inf	168.	34.621	{13.5158, 53.0825}	79.3923	0.738398	newly	85.	60.5	{24., 96.}	35.4588	0.232112	Reff	0.583642	0.592611	{0.585026, 0.598143}	1.55391	ar	6842.	6550.06	{6496., 6604.}	4.26688
39	R0	{1.66621, 11.4824, 0.1}	{1.65176, 2.31647, 1.18}	{{1.61068, 2.12856, 0.}, {1.67287, 2.78994, 4.}}	{1.42695, 79.8259, 1220.}	inf	221.	42.8664	{14.9404, 66.1549}	80.6034	0.771169	newly	130.	82.05	{27., 127.}	39.9615	0.276932	Reff	0.657653	0.681379	{0.661793, 0.694458}	3.86625	ar	6473.	5957.71	{5866., 6045.}	7.96061
40	R0	{1.60694, 9.95741, 1.}	{1.64158, 3.18553, 1.2}	{{1.61068, 2.78994, 0.}, {1.65456, 3.37285, 4.}}	{2.15563, 68.0084, 160.}	inf	179.	37.4886	{19.458, 55.8011}	79.0566	0.72421	newly	116.	55.06	{27., 82.}	52.5345	0.370068	Reff	0.592157	0.628146	{0.616456, 0.634803}	6.07759	ar	6625.	6235.23	{6179., 6290.}	5.88332
41	R0	{1.55128, 4.71911, 0.1}	{1.55606, 4.84218, 3.01}	{{1.55618, 4.83753, 3.}, {1.55618, 4.83753, 3.}}	{0.31669, 2.60797, 2910.}	inf	16.	23.8424	{13.9242, 34.18}	55.793	0.186036	newly	16.	26.31	{14., 38.}	70.6875	0.219866	Reff	0.605309	0.605954	{0.604184, 0.607442}	0.196094	ar	6141.	6141.06	{6112., 6176.}	0.335125
42	R0	{1.60063, 6.81118, 0.1}	{1.65865, 5.3704, 5.}	{{1.65865, 5.3704, 5.}, {1.65865, 5.3704, 5.}}	{3.62488, 21.1532, 4900.}	inf	61.	23.7224	{15.0554, 34.4125}	61.1108	0.435251	newly	44.	24.14	{14., 36.}	45.2727	0.292172	Reff	0.565983	0.592682	{0.590909, 0.594103}	4.71733	ar	6528.	6459.03	{6431., 6489.}	1.06173
43	R0	{1.56986, 5.44652, 0.1}	{1.59034, 5.09935, 4.}	{{1.59034, 5.09935, 4.}, {1.59034, 5.09935, 4.}}	{1.30475, 6.37419, 3900.}	inf	36.	18.9519	{9.33819, 27.412}	47.3558	0.314098	newly	40.	20.24	{10., 29.}	49.4	0.333412	Reff	0.586343	0.596704	{0.595306, 0.598057}	1.76715	ar	6312.	6276.65	{6249., 6306.}	0.601553
44	R0	{1.47523, 8.07785, 0.1}	{1.46417, 4.14223, 0.65}	{{1.43965, 2.60601, 0.}, {1.4804, 6.00596, 2.}}	{0.975818, 48.7212, 674.}	inf	154.	44.7886	{26.6908, 60.3536}	70.9165	0.559425	newly	118.	61.62	{31., 94.}	47.8305	0.3159	Reff	0.674918	0.685845	{0.675299, 0.695212}	1.65353	ar	5692.	5411.11	{5339., 5477.}	4.93482
45	R0	{1.6417, 7.69429, 0.1}	{1.67405, 7.28757, 1.}	{{1.67405, 7.28757, 1.}, {1.67405, 7.28757, 1.}}	{1.97049, 5.28594, 900.}	inf	70.	154.384	{139.222, 170.835}	120.549	0.342288	newly	45.	120.21	{104., 137.}	167.133	0.424275	Reff	0.547999	0.544671	{0.541891, 0.547161}	0.613464	ar	6735.	6923.33	{6869., 6965.}	2.79629
46	R0	{1.38865, 5.83473, 0.1}	{1.45066, 3.97559, 0.01}	{{1.44762, 3.83841, 0.}, {1.47488, 4.00571, 0.}}	{4.46544, 31.8633, 108.}	inf	81.	45.3183	{24.0896, 66.6929}	44.7085	0.290591	newly	88.	62.38	{32., 91.}	33.7727	0.205576	Reff	0.6778	0.713243	{0.708655, 0.722521}	5.22912	ar	5233.	5190.51	{5097., 5280.}	1.22435
47	R0	{1.45289, 6.87903, 0.1}	{1.44485, 6.00596, 0.}	{{1.44485, 6.00596, 0.}, {1.44485, 6.00596, 0.}}	{0.553564, 12.6917, 100.}	inf	113.	66.2139	{50.5304, 82.3342}	41.4036	0.239181	newly	105.	64.48	{47., 85.}	38.5905	0.221918	Reff	0.690704	0.69364	{0.691229, 0.695873}	0.435297	ar	5379.	5340.88	{5271., 5405.}	1.02435
48	R0	{1.69416, 9.4831, 0.1}	{1.83592, 11.1369, 0.}	{{1.83592, 11.1369, 0.}, {1.83592, 11.1369, 0.}}	{8.3678, 17.4392, 100.}	inf	191.	396.121	{377.641, 412.71}	107.393	0.316486	newly	108.	217.28	{197., 234.}	101.185	0.302755	Reff	0.561783	0.571134	{0.567899, 0.574491}	1.66439	ar	6925.	7338.92	{7267., 7407.}	5.97718
49	R0	{1.47843, 7.16286, 0.1}	{1.44494, 6.00246, 0.03}	{{1.44485, 6.00596, 0.}, {1.44485, 6.00596, 0.}}	{2.26532, 16.2002, 128.}	inf	84.	59.4266	{44.7209, 76.4607}	29.281	0.159167	newly	57.	57.98	{43., 76.}	18.5614	0.0817535	Reff	0.694419	0.682239	{0.679677, 0.68432}	1.75398	ar	5449.	5400.53	{5335., 5475.}	1.11268
50	R0	{1.68843, 5.72427, 0.1}	{1.69756, 6.15907, 2.}	{{1.69756, 6.15907, 2.}, {1.69756, 6.15907, 2.}}	{0.541033, 7.59571, 1900.}	inf	27.	55.764	{44.4632, 65.3739}	106.533	0.310704	newly	30.	49.61	{39., 61.}	65.4333	0.212445	Reff	0.541311	0.539356	{0.537704, 0.541208}	0.37527	ar	6800.	6888.46	{6856., 6925.}	1.30088
